# Racial and Ethnic Variation in Genetic Susceptibility: Are Disparities in Infertility Prevalence and Outcomes more than Black and White?

**DOI:** 10.1007/s43032-022-00956-5

**Published:** 2022-04-28

**Authors:** Jerrine R. Morris, Torie Comeaux Plowden, Lisa J. Green, Digna R. Velez Edwards, Tia Jackson-Bey

**Affiliations:** 1 Department of Obstetrics, Gynecology, and Reproductive Sciences, University of California San Francisco, 499 Illinois Street, San Francisco, CA 94158 USA; 2 Department of Obstetrics and Gynecology, Walter Reed National Military Medical Center, Bethesda, MD USA; 3 Department of Obstetrics and Gynecology, School of Medicine, University of South Carolina, Greenville, SC USA; 4 Fertility Center of the Carolinas-Greenville, Greenville, SC USA; 5 Division of Quantitative Sciences, Department of Obstetrics and Gynecology, Vanderbilt University Medical Center, Nashville, TN USA; 6 Department of Biomedical Informatics, Vanderbilt University Medical Center, Nashville, TN USA; 7 Reproductive Medicine Associates of New York, New York, NY USA; 8 Department of Obstetrics and Gynecology and Reproductive Science, Division of Reproductive Endocrinology and Infertility, Mount Sinai School of Medicine, New York, NY USA

**Keywords:** Genetic ancestry, Health disparities, Infertility, Heritability

## Abstract

Race, as a social construct without a clear genetic underpinning, is frequently referenced in medicine as predictor of multiple diseases including that of infertility. The authors will discuss how systematic racism can have downstream consequences ranging from overt physician bias to use of medical algorithms that may potentiate the same disparities they attempt to narrow. Then, the authors explore the utility and pragmatic use of genetic ancestry to estimate disease prevalence, instead of racial categories. Finally, the authors explore how health inequities, rooted in systematic racism, can influence disease heritability effectively advocating for research to disentangle the contributions of racism to genetic susceptibility in infertility.

As recently as 2020, the American Medical Association (AMA) voted to adopt policies underscoring race as a socially constructed category [1]. This acknowledgment is in response to mounting evidence that race is not an inherent biological trait and thus disparities in adverse health outcomes are due, to a large extent, to systemic racism and/or stressors resulting from racism [1]. Systemic racism is the normalization and legitimization of a multitude of behaviors and factors that stem from historical, cultural, institutional, and interpersonal sources that tend to advantage White people while perpetuating or worsening adverse outcomes for people of color. As the medical community learns more about the impact of the social determinants of health, these discoveries have challenged many of our traditionally held beliefs and dogmas related to race and its impact on health. What is the utility, if any, of referencing race in medicine, in women’s health, and, more specifically, in infertility?

When providers use race or ethnicity to withhold or alter treatment, whether consciously or not, they become conduits of the racism that perpetuates disparities in medicine. Overt racism is less accepted in modern medicine; however, false beliefs of biological differences based on race often conceal unconscious bias and perpetuate the influence of racism in medicine. Hoffman et al. found at least 50% of residents and medical students held at least one false belief regarding biologic differences between White and Black Americans. Medical trainees with a greater number of false beliefs were less likely to recommend the appropriate treatment for Black patients in this fictional case study [2]. Direct consequences are seen in women’s reproductive health. The estimated prevalence of endometriosis is higher among White and Asian women and lower among Black women [3]. However, Black women are disproportionately treated for (presumptive) pelvic inflammatory disease (PID) when presenting with chronic pelvic pain instead of considering the full spectrum of pelvic pain etiologies, including endometriosis. Thus, many Black women with endometriosis may have a delayed diagnosis because they were thought to have PID. The utilization of race by providers often enforces false narratives and implicit biases that disenfranchise persons of color.

In an era of increased algorithms, tools to quickly identify patients who may be at risk for an adverse outcome have emerged. Several of these tools utilize race and ethnicity to determine risk stratification, which has in many cases resulted in an inadvertent disparity of healthcare delivery. The most notable in the field of women’s health is the vaginal birth after cesarean (VBAC) risk calculator. When used, self- or provider-reported Black race or Hispanic ethnicity decreased one’s probability of a successful live birth. Thus, these women would be less likely offered the opportunity for VBAC. This decrement was comparable to the net benefit one could gain with a history of a prior vaginal delivery or VBAC [4]. Similarly, the National Cancer Institute Breast Cancer Risk Assessment Tool, which is used to estimate a woman’s risk of developing invasive breast cancer in the next 5 years, includes race as a variable. While “validated” within different racial and ethnic populations, lower risk estimates are provided for all racial and ethnic minority women when compared to White women. This algorithm may falsely reassure providers when seeing women of color leading to inadequate screening in nonwhite women [4]. Use of such inherently biased algorithms to counsel and/or treat diverse patients often perpetuate rather than ameliorate disparities in women’s health.

Accepting these limitations yet understanding the desire for evidence-based treatment algorithms, is there ever a time where race/ethnicity should be considered? The answer to this is — well — yes. However, instead of highlighting racial and ethnic differences, the focus should be shifted towards identifying ancestral markers that influence disease prevalence. The limiting factor in the use of race as a surrogate marker for genetic ancestry is that in most studies, including those reflected in reproductive medicine, race is self-reported. Kaseniit et al. found self-reported race was an imperfect proxy for genetic ancestry as roughly (only) 9% of patients who underwent genetic testing were found to have concordance between their genetic ancestry and self-reported race. Concordance was lowest among those who self-reported Middle Eastern, Ashkenazi Jewish, and Southern European descent [5]. Furthermore, these algorithms seldomly originate from nonwhite populations. In reproductive medicine, much of the current research is focused on ways to optimize outcomes in in vitro fertilization (IVF) when treating infertility. Preimplantation genetic testing is a technology which has been employed for this very reason — proponents of this technology argue for its use to detect structural chromosomal abnormalities, reduce heritability of single gene disorders, and potentially limit transfer of chromosomally abnormal embryos [6]. A new technology has since emerged — preimplantation testing with polygenic risk scores (PRSs) [7]. While the opportunity to potentially rank-order euploid embryos may seem advantageous, these genome-wide association studies have only been validated using European ancestry thus extrapolating risks may not be valid for populations without this shared ancestry [8]. Hence, even when genetic ancestry is used in algorithms, exclusive validation using European ancestry will still limit their clinical applicability and may further widen disparities in nonwhite populations.

Certain diseases like endometriosis and PCOS have increased heritability within families but how it applies to self-reported racial groups may need to be reexamined. Other reproductive health diagnoses, such as uterine fibroids, have stronger ties along self-reported racial groups, but this is more likely a consequence of genetic ancestry than physical attributes of race. Keaton et al. investigated genetic ancestry proportions for populations clustered into six geographic groups and found northern European ancestry to be protective against fibroids while west African ancestry typically conferred increased risk of fibroid prevalence among Black and White women [9]. Similarly, genome-wide association studies have explored single nucleotide polymorphisms (SNPs) associated with age at menopause. Japanese, Chinese, and African American women have all exhibited SNPs that were not implicated (seen/observed/demonstrated) in European populations [10].

Finally, we are only just beginning to understand the degree to which environmental exposures influence transgenerational health. Diethylstilbestrol (DES) is an endocrine disruptor that is a well-known transplacental pathogen. Exposure in utero leads to increased risks for reproductive tract anomalies, infertility, and clear cell adenocarcinoma. However, third-generation women exposed have been shown to have an increased risk of preterm birth and menstrual irregularities as compared to their counterparts, suggesting long-term effects that permeate multiple generations [11]. Through use of a rat model, studies have shown how in utero exposure to 2,3,7,8-tetrachlorodibenzo-p-dioxin (TCDD), a common pollutant found in solid waste and often a contaminant of food products, can increase risk of reduced fertility and preterm birth among future offspring [12]. Epigenetic alterations have been proposed as the link between the effects of environmental exposures on poor reproductive health outcomes. In another example, US borne Black women have higher rates preterm birth (PTB) as compared to both Foreign borne non-Hispanic Black women and White women [13, 14]. Vitamin D deficiency has been shown to be associated with spontaneous PTB. Interestingly, transcriptomic analyses have found overlapping gene dysregulation during both vitamin D deficiency and PTB [15]. One can postulate how disparate access to resources that often affect minority populations may incorrectly lead one to suspect a “genetic” cause of adverse reproductive health outcomes when, in fact, this transgenerational morbidity is a result of systemic racism.

Health inequities exist in a complex, multi-factorial environment and are related to differences in access to care, differences in resources that promote health, and yes, due to the impact of systematic racism that is pervasive in the USA. Simply reporting health disparities is not enough anymore—arguably, it was never enough. Research needs to focus on how genetic ancestry may affect a population’s disease susceptibility and can be effectively and equitably used to identify prevention and treatment strategies. Tools to validate one’s risk using ancestral markers must originate from unique populations to increase their generalizability and subsequent clinical utility. Meanwhile, all fields of medicine must take a stark look at systematic inequities and work to dismantle them. Now is the time to move beyond our traditional categorizations of humans, steeped in division and hierarchy, and to some extent rooted in white supremacy and patriarchy, to discover new paradigms for improving health and medical care for all.

## Data Availability

Not applicable.
